# Evaluating the psychometric properties of the 24-item and 12-item real relationship inventory-client forms

**DOI:** 10.1371/journal.pone.0311411

**Published:** 2025-03-03

**Authors:** Alberto Stefana, Paolo Fusar-Poli, Eduard Vieta, Eric A. Youngstrom

**Affiliations:** 1 Department of Psychiatry and Behavioral Health, The Ohio State University, Columbus, Ohio, United States of America; 2 Department of Brain and Behavioral Sciences, University of Pavia, Pavia, Italy; 3 OASIS Service, South London and Maudsley NHS Foundation Trust, London, United Kingdom; 4 Early Psychosis: Interventions and Clinical-detection (EPIC) Lab, Department of Psychosis Studies, Institute of Psychiatry, Psychology & Neuroscience, King’s College London, London, United Kingdom; 5 Bipolar and Depressive Disorders Unit, Hospital Clinic, IDIBAPS, CIBERSAM, Institute of Neuroscience, University of Barcelona, Barcelona, Catalonia, Spain; 6 Institute for Mental and Behavioral Health Research, Nationwide Children’s Hospital and Department of Psychiatry, The Ohio State University, Columbus, Ohio, United States of America; 7 Helping Give Away Psychological Science, Chapel Hill, North Carolina, United States of America; Fayoum University Faculty of Education, EGYPT

## Abstract

The current study assessed the psychometric properties of the long (24 items) and brief (12 items) versions of the Real Relationship Inventory–Client (RRI-C) in a United States sample. The RRI-C is the most used quantitative measure of the real relationship construct, yet its psychometric properties have not been explored outside its development studies. A sample of 700 adults in individual psychotherapy was recruited in the study and filled out a comprehensive battery of measures. Analytical techniques included confirmatory factor analysis (CFA), exploratory structural equation modeling (ESEM), multigroup CFA, multigroup factor analysis alignment, item response theory, internal reliability assessments, Bland-Altman regression analysis, and calculation of reliable change benchmark thresholds. Both RRI-C versions demonstrated a bifactor structure encompassing Genuineness and Realism dimensions. The bifactor ESEM model provided strong fit: χ^2^_[210]_ =  482.464, CFI =  0.999, TLI =  0.998, RMSEA =  0.043, SRMR =  0.020 for the 24-item RRI-C; χ^2^_[45]_ =  111.916, CFI =  0.999, TLI =  0.998, RMSEA =  0.046, SRMR =  0.028 for the 12-item RRI-C. McDonald’s omega total was 0.97 and 0.95 respectively. The correlation between the total scores of the two versions was *r* =  0.98; the average discrepancy was 1.85 points higher for the comprehensive version with a slope of -0.013 (*p* =  0.12). Both versions showed functionally identical reliability and factor structure when therapy is online *vs.* in-person. Significant correlations were found between the RRI-C and the Working Alliance Inventory (*r* =  0.68 and *r* =  0.67 for the 24-item and 12-item versions, respectively, both *p* < .001) and the Session Evaluation Scale (*r* =  0.62 and *r* =  0.58, respectively, both *p* <  0.001). This study substantiates the sound psychometric properties of the 24-item and 12-item RRI-C.

## Introduction

Since the inception of psychotherapy, the authentic connection between therapists and patients—known as the real relationship—has been acknowledged as a crucial aspect of the therapeutic process [1]. This genuine interaction, marked by a real relationship and an accurate understanding of each individual, surpasses the theoretical boundaries of therapy [2,3]. The strength of the real relationship is determined by the degree and nature of its primary components: realism and genuineness. Realism embodies the actual experience and understanding of the other, whereas genuineness signifies the sincerity expressed towards the other. These components highlight the distinct traits of each participant and the quality of their relationship [2,4]. The creation and robustness of this interpersonal entity depend on the combined efforts of both the therapist and the patient [5].

Although all elements of the therapeutic relationship likely intertwine [6], the alliance, transference, and attachment seem to be especially important for the real relationship [7]. The real relationship, a nonworking connection, and the working alliance, a working connection, are distinct yet deeply interlinked constructs that maintain a similar relationship. Recent meta-analytical evidence supports this, showing a high positive correlation [8]. Transference, however, exhibits a modest and negative correlation with the real relationship [5,9]. Regarding attachment, patients and therapists with secure attachment styles, characterized by low anxiety and avoidance attachment, tend to cultivate a stronger real relationship [1,10,11].

Notably, meta-analytical findings indicate a moderate correlation between the strength of the real relationship and the psychotherapy outcomes, regardless of the type of outcome or the source of information [12]. This correlation surpasses those observed in the alliance-outcome relation in recent meta-analyses (ranging from *r* = .20 to.29) [13–15].

Over the last 30 years, most quantitative research on the real relationship has used the Real Relationship Inventory–Therapist Version (RRI-T) [16] and the Real Relationship Inventory–Client Version (RRI-C) [17]. The 12-item shorter versions were adapted from these inventories by rational selection of items believed to best represent the realism and genuineness aspects [18]. However, these condensed versions were not subject to comprehensive psychometric validation [19,20].

The objective of this study was to conduct a thorough assessment and comparison of the psychometric characteristics of the 24-item and 12-item versions of the RRI-C within a US-based sample of individuals undergoing individual psychotherapeutic treatment. Specifically, we aimed to enhance the literature by testing their factor structure. This was achieved by employing exploratory structural equation modeling, confirmatory factor analysis, multigroup confirmatory factor analysis, and multigroup factor analysis alignment. We used item response theory models to assess item characteristics and to ensure a range of good score reliability. Additionally, McDonald’s omega was employed as a more reliable estimate for tools with multiple factors or subscales [21,22]. Furthermore, the Bland-Altman methods [23] were used to evaluate the calibration and consistency of scores when comparing short versus full-length forms. The findings from this comprehensive suite of methods could boost confidence in both the short and full-length forms. They also provide insights into clinical situations where scores might warrant less confidence. We also develop provisional benchmarks for clinical change and process milestones, to facilitate clinical applications.

## Materials and methods

### Study design

This article presents secondary analyses of the baseline data from a longitudinal study [24]. The research was approved by the Institutional Review Board of the University of North Carolina at Chapel Hill (Study #: 23-0216; Approval Date: 3/06/2023).

### Participants

The study involved 700 adult patients who participated in different types of individual therapy for various psychological disorders. Females made up 81% (*n* =  564) according to biological sex, and 74% (*n* =  512) in terms of gender self-identification. The most prevalent age brackets were those aged 23-29 years (20%, *n* =  142) and 30-39 years (28%, *n* =  193). Most of the participants (81%, *n* =  566) were of white ethnicity and had at least one diagnosed psychiatric disorder (84%, *n* =  590), with anxiety (66%, *n* =  464) and unipolar depression (56%, *n* =  391) being the dominant conditions. More than half of participants attended their most recent therapy session via video call (53%, *n* =  369). The rest were in-person face-to-face session (36%, *n* =  251), telephone call (8%, *n* =  51

9), and in-person session on the couch (3%, *n* =  21). Table 1 shows a comprehensive view of the demographics, clinical histories, and therapeutic characteristics of the sample.

**Table 1:** Demographics, clinical, and treatment characteristics of the participating patients (*N* = 700).

Demographics	% (*n*)
Age (years)	
18–22	9% (66)
23–29	20% (142)
30–39	28% (193)
40–49	16% (109)
50–59	14% (99)
≥60	13% (91)
Biological sex	
Female	81% (564)
Male	18% (128)
Intersex	0% (1)
I prefer not to say	1% (7)
Gender	
Woman	74% (512)
Man	19% (132)
Other	4% (29)
Woman–Other	2% (14)
Man–Other	0% (3)
I prefer not to say	1% (6)
Education	
Less than high school	0% (2)
High school graduate	3% (24)
Some college	19% (136)
2-year degree	9% (64)
4-year degree	33% (231)
Professional degree	28% (195)
Doctorate	7% (48)
Ethnicity	
White	81% (566)
Black or African American	10% (68)
Asian	4% (29)
Native Hawaiian or Pacific Islander	1% (4)
American Indian or Alaska Native	1% (5)
Other	4% (28)
Clinical characteristics a	
Any psychiatric disorder	84% (590)
Any anxiety disorder	66% (464)
Any (unipolar) depressive disorder	56% (391)
Any trauma- and stressor-related disorders	35% (244)
Any neurodevelopmental disorder	24% (165)
Any bipolar or related disorder	13% (88)
Any eating disorder	10% (71)
Any disruptive behavior and dissocial disorder	2% (15)
Schizophrenia or any other psychotic disorders	1% (9)
Any cluster A personality disorder	0% (3)
Any cluster B personality disorder	6% (43)
Any cluster C personality disorder	6% (41)
Treatment characteristics	
In psychotherapy from	
0 to 3 months	14% (99)
4 to 6 months	14% (96)
7 to 12 months	11% (79)
13 to 24 months	13% (94)
>24 months	47% (332)
Session frequency	
1 or less per month	19% (130)
2 to 3 per month	39% (276)
1 per week	38% (267)
2 or more per week	4% (27)
Session attendance	
Video call	53% (369)
In person face to face	36% (251)
Telephone call	8% (59)
In person on the couch	3% (21)
Therapy location	
Private practice	70% (493)
Private health institution	11% (76)
Public health institution	10% (67)
University counseling center	4% (26)
Other	5% (38)
Therapist biological sex (Female)	81% (565)

*Note.* ^a^ *N* sums to more than 700 because cases could have more than one diagnosis.

### Measures

#### Sociodemographic and clinical domain.

A patient data form was administered to collect the sociodemographic, clinical, and treatment data reported in Table 1.

#### Personality domain.

The *Big Five Inventory–2-Extra-Short form* (BFI-2-XS) [25] is a 15-item scale that assesses the five main personality domains. Each item is rated on a 5-point Likert scale. In our study, the internal consistency for this measure ranged 0.50–0.65 for various dimensions.

The *Level of Personality Functioning Scale-Brief Form 2.0* (LPFS-BF 2.0) [26] is a 12-item self-report questionnaire that assesses the severity of symptoms of personality disorder according to the DSM-5 Section III criteria. The tool uses a 5-point Likert scale for ratings, generating a Cronbach’s *α* of 0.85 in our study.

#### Mental health state domain.

The *Generalized Anxiety Disorder-7* (GAD-7) [27] is a self-administered questionnaire for assessing the prevalence and severity of generalized anxiety disorder. The tool consists of seven items and exhibited a reliability of *α* =  0.88 in our study.

The *Patient Health Questionnaire-9* (PHQ-9) [28] is a 9-item self-reporting measure designed to evaluate the severity of depressive symptoms, with a 4-point Likert scale used for responses. In our study, the internal consistency of the PHQ-9 was *α* =  0.86.

The *International Positive and Negative Affect Schedule - Short Form* (I-PANAS-SF) is a 10-item scale that measures both positive and negative affective states over the past week. It used a five-point Likert scale, and in our sample, the Positive Affect and Negative Affect scales showed coefficients of *α* =  0.78 and 0.74, respectively.

The *Single-item global measures of symptom severity, psychosocial functioning, and quality of life* [29] consist of three single items that evaluate severity of symptoms, psychosocial functioning, and quality of life. All items use a 5-point Likert scale for responses. No internal consistency is reported for single item scales.

#### Therapeutic relationship domain.

The *Real Relationship Inventor–Client Form* (RRI-C) [17] is a 24-item self-report measure rooted in solid theoretical foundations. It gauges the patients’ perception of the strength of their real relationship with their therapist, focusing on two significant constructs: genuineness and realism. The process of item analysis and expert validation distilled an initial pool of 180 items to 24. These items, distributed on two subscales, are rated on a Likert scale from 1 (“Strongly disagree”) to 5 (“Strongly agree”). Eight of the items (#3, 6, 8, 12, 14, 21, 22, and 24) are reversed keyed before scoring. The RRI-C development and validation stages prioritized theoretical congruence, with item selection emphasizing alignment with the constructs of genuineness and realism. Subsequent confirmatory factor analysis supported the two-factor model, reinforcing these constructs as central elements in the therapeutic relationship. Although robust psychometric properties were demonstrated, the development process prioritized the theoretical approach. Subsequently, the RRI-C was streamlined into a succinct 12-item measure [18], composed of the Realism and Genuineness subscales with six items each. The authors handpicked these 12 items, considering them to be the best representatives of the two constructs within the longer measures, signifying the continued focus on the theoretical representation in the measures. Internal consistency estimates were Cronbach’s *α* of.91 for the scale evaluating Genuineness, 0.90 for Realism, and 0.95 for the total score.

The *Working Alliance Inventory–Short Revised* (WAI-SR) [30] is a 12-item questionnaire designed to assess the strength of the working alliance in therapeutic settings. It includes three subscales that investigate: (a) alignment on therapeutic tasks, (b) agreement on therapeutic goals, and (c) development of an emotional bond between the therapist and patient. Responses are evaluated on a six-point Likert scale (0 to 5). The tool exhibited a Cronbach’s *α* of 0.95 in our study.

The *Section B of the Post-Session Questionnaire* (PSQ) [31] comprises 4 items assessing alliance ruptures and their resolution during a therapy session. In the event of a conflict, the next three items measure the peak tension level, the degree to which the issue was addressed, and the resolution level from the patient’s perspective. Because of the gated nature of the second set of items, internal consistency is not reported.

The *Rift In-Session Questionnaire* (RISQ) [32] is a 4-item questionnaire that evaluates potential rifts in the therapeutic relationship, as indicated by feelings of rejection, belittlement, and thoughts of disobedience. It used a binary response format (Yes/No), with higher scores suggesting a higher risk of rifts. In our sample, it showed a Cronbach’s α of 0.71.

The *in-Session Patient Affective Reactions Questionnaire* (SPARQ) [32,33] is an 8-item self-administered tool measuring patient’s affective responses and perceptions toward their therapist during therapy. The two scales, Positive Affect and Negative Affect, reflect the patient’s sense of security in the therapeutic relationship and feelings of worry, shame, and need for help. A 5-point Likert scale is used for the ratings, with a Cronbach’s α of 0.86 and 0.75 for Positive Affect and Negative Affect scales, respectively, in our study.

#### Session outcome domain.

The *Session Evaluation Scale* (SES) [34,35] is a 5-item self-report scale developed to assesses a patient’s perception of a therapy session’s quality. It uses a 5-point Likert scale, and the final score is calculated by averaging the individual item scores (after appropriate reversals). The instrument demonstrated a reliability of α =  0.86 in our study.

### Statistical analyses

The suitability of the data for factor analysis was initially verified using the Kaiser-Meyer-Olkin (KMO) [36] test and the Bartlett sphericity test [37].

Following this, we analyzed the factor structure of the RRI-C. Initially, we applied confirmatory factor analysis (CFA) with maximum likelihood (ML), to assess the original two-factor model suggested by the authors. Our goal was to confirm whether our data fit this established measurement model, thereby supporting the validity of our research findings. Following this, due to significant correlations between subscales and cross-loadings of items, we conducted a CFA to evaluate a one-factor model. Subsequently, we employed an exploratory structural equation modeling (ESEM) to test the original two-factor model. ESEM approach synergizes elements of exploratory factor analysis and CFA, facilitating confirmatory tests for predetermined factor structures [38]. A distinctive feature of ESEM is its ability to accommodate cross-loadings, allowing items to load on multiple factors. It effectively constrains non-primary item-factor associations to approximately zero, thereby preventing inflated parameter estimates or distorted model fit. In this study, both Geomin and targeted rotations were employed. The Geomin rotation adopts an exploratory stance, setting a predefined number of latent factors and enabling the algorithm to discern the primary loading items for each factor [39]. On the other hand, the targeted rotation is more hypothesis-driven, allowing for the integration of cross-loadings within the framework of a hypothesized model. This method considers targeted items in the context of both their primary dimension and other relevant dimensions. Such a dual approach in ESEM enhances the robustness and specificity of the factor structure analysis in the context of the RRI-C. Finally, we used both CFA and ESEM to examine a bifactor model.

During the model fit evaluation, we used the Comparative Fit Index (CFI) [40], Tucker–Lewis Index (TLI) [41], Root Mean Square Error of Approximation (RMSEA) [42], and Standardized Root Mean Squared Residual Index (SRMR) [43]. We adhered to conventional thresholds recommended to signify a well-fitting model: CFI and TLI values of 0.95 or above and RMSEA and SRMR values of 0.08 or below [43,44].

As a subsequent step, we employed a multi-group CFA with maximum likelihood method to test the measurement invariance of the RRI-C between in-person and online format settings. The multi-group CFA process encompassed three stages of testing equivalence with escalating restrictions: configural (unconstrained), metric (fixed factor loadings), and scalar (fixed factor loadings and intercepts). The determination of invariance was executed by observing alterations in the fit indices: changes in CFI and TLI below 0.01, RMSEA below 0.015, and SRMR below 0.03 are considered acceptable [45]. Because the classic approach to multi-group CFA does not allow for estimating the effect size of item bias, we used the effect size measure for differences in mean and covariance structures (*d*_MACS_) to evaluate the magnitude of measurement invariance at item level [46]. In addition, given that satisfying scalar invariance is often challenging in clinical research, we performed a multi-group factor analysis alignment. This method offers a more practical alternative for testing metric and scalar invariances because it does not impose equality restrictions on factor loadings or intercepts between groups [47]. Consequently, if demonstrating complete metric and scalar invariance between in-person and online session formats is unattainable using traditional multi-group CFA, a less stringent method will be employed. We implemented the alignment procedure through a fixed method, setting alignment power values for lambda (*λ*) (loadings) and nu (*ν*) (intercepts) at 0.25, with lambda and nu tolerances established at 0.4 and 0.2, respectively [48].

Additionally, we employed a Multiple Indicator Multiple Cause (MIMIC) model to test for measurement invariance adjusted for age and gender between assessment types. We also used robust chi-squared difference tests using the Satorra-Bentler correction to assess the impact of these demographic factors on the measurement structure across the groups.

Following factor analyses, item response theory (IRT) graded response model (GRM) proposed by Samejima [49] was used to examine the psychometric properties of each item belonging to the original Genuineness and Realism factors. The GRM was selected because it is well-suited for analyzing Likert-scale data, providing a detailed assessment of item discrimination and difficulty. The GRM allows each item to have a unique relationship with a latent trait, thereby calculating the item discrimination (*a*) and difficulty (*β*) parameters. The *a* value indicates the sensitivity of an item at a certain difficulty level represented by *β*. The steeper the scale slope, the better the item differentiates between different levels of the trait [50]. The *β* value represents a point at which a respondent with an equivalent ability level to a given difficulty level has a 50% probability of responding at or above that difficulty level.

Internal consistency was evaluated using Cronbach’s alpha [51], the correlation of average items, and McDonald’s omega total (*ω*) [52]. Additionally, IRT estimated marginal reliability at various theta levels [53]. The projected reliability and content coverage for the short version of the scale were calculated using the formulas from Smith et al. [54].

To assess the precision of scores obtained from each of the RRI short forms compared to the comprehensive 24-item version, Bland-Altman plots were used [55]. These plots provided evaluations of score bias and the “limits of agreement,” denoted by the standard deviation of score discrepancies (here, used to construct a 95% confidence interval).

Lastly, the criterion validity of the short-form scale was established by examining its correlations with patient demographic and clinical characteristics, validated measures of personality traits, current mental health state, and specific elements of the therapeutic relationship. The correlations between our scale and these validated measures highlight our scale’s practical utility in both clinical and research settings.

We used the packages lavaan 6-11 [56] (CFA), esem 2.0.0 [57] (for ESEM), sirt 3.13-228 [58] (analysis alignment), mirt 1.36.1 [59] (IRT analysis), psych 2.2.9 [60] (scoring, classical test theory reliability estimates), and ggplot2 3.4.2 [61] and semPlot 1.1.6 [62] (additional visualizations) implemented in the R Software and Programming environment 4.3.1.

### Procedure

Participants were recruited from two online patient registers, ResearchMatch and Research for Me, during March and April 2023. ResearchMatch, which boasts a volunteer base of more than 158,000, is a collaborative effort by leading academic institutions and is supported by the Clinical and Translational Science Awards (CTSA) Program of the United States National Institutes of Health. Research for Me, which hosts more than 24,000 volunteers, is an initiative of the NC TraCS Institute at the University of North Carolina at Chapel Hill and is also part of the CTSA program. Previous studies have shown that participants recruited through online platforms tend to provide reliable data, especially in terms of accurate demographic and psychological information, without the influence of financial incentives [63]. The study eligible participants were adults 18 years and older, engaged in individual psychotherapy, fluent in English, and able to give informed consent. Following their consent, the participants underwent an initial evaluation focusing on their most recent therapy session and the experiences of the previous week, using Qualtrics software for the survey.

## Results

### Items descriptive statistics

Table 2 presents descriptive statistics for each item of the RRI-C. S1 Fig provides histograms of the distribution of responses for each item.

**Table 2:** Descriptive statistics of the RRI-C items.

Item n.	Item content	Dim	Mean	SD	Median	Trimmed	MAD	range	skew	kurtosis	SE
1	I was able to be myself with my therapist	G	4.12	1.17	4.5	4.36	0.74	1–5	−1.41	1.11	0.04
2	My therapist and I had ^a^ realistic perception of our relationship	R	4.03	1.11	4	4.23	1.48	1–5	−1.25	0.98	0.04
3	I was holding back significant parts of myself ^R^	G	3.65 ^a^	1.27	4	3.79	1.48	1–5	−0.67	−0.67	0.05
4	I appreciated being able to express my feelings in therapy	G	4.23	1.08	5	4.46	0.00	1–5	−1.58	1.90	0.04
5	My therapist liked the real me	R	3.86	1.01	4	3.96	1.48	1–5	−0.78	0.37	0.04
6	It was difficult to accept who my therapist really is ^R^	R	4.10	1.10	4	4.31	1.48	1–5	−1.28	0.93	0.04
7	I was open and honest with my therapist	G	4.08	1.07	4	4.29	1.48	1–5	−1.34	1.27	0.04
8	My therapist’s perceptions of me seem colored by his or her own issues ^R^	R	4.01	1.21	4	4.21	1.48	1–5	−1.08	0.05	0.05
9	The relationship between my therapist and me was strengthened by our understanding of one another	R	3.78	1.03	4	3.90	1.48	1–5	−0.76	0.22	0.04
10	My therapist seemed genuinely connected to me	G	3.87	1.07	4	4.01	1.48	1–5	−0.90	0.21	0.04
11	I was able to communicate my moment-to-moment inner experience to my therapist	G	3.80	1.07	4	3.91	1.48	1–5	−0.74	−0.2	0.04
12	My therapist was holding back his/her genuine self ^R^	G	3.84	1.05	4	3.96	1.48	1–5	−0.73	−0.14	0.04
13	I appreciated my therapist’s limitations and strengths	R	3.89	0.95	4	3.99	1.48	1–5	−0.78	0.40	0.04
14	We do not really know each other realistically ^R^	R	3.24	1.19	3	3.29	1.48	1–5	−0.25	−0.94	0.05
15	My therapist and I were able to be authentic in our relationship	G	3.88	1.01	4	4.02	1.48	1–5	−0.94	0.54	0.04
16	I was able to see myself realistically in therapy	R	3.91	1.00	4	4.04	1.48	1–5	−0.91	0.39	0.04
17	My therapist and I had an honest relationship	G	4.03	1.03	4	4.20	1.48	1–5	−1.18	0.95	0.04
18	I was able to separate out my realistic perceptions of my therapist from my unrealistic perceptions	R	3.86	0.96	4	3.97	1.48	1–5	−0.79	0.35	0.04
19	My therapist and I expressed ^a^ deep and genuine caring for one another	G	3.45	1.09	4	3.50	1.48	1–5	−0.35	−0.52	0.04
20	I had ^a^ realistic understanding of my therapist as ^a^ person	R	3.73	1.02	4	3.84	1.48	1–5	−0.88	0.38	0.04
21	My therapist did not see me as I really am ^R^	R	3.85	1.14	4	4.01	1.48	1–5	−0.97	0.16	0.04
22	I felt there was ^a^ significant holding back in our relationship ^R^	G	3.83	1.16	4	3.97	1.48	1–5	−0.83	−0.25	0.04
23	My therapist’s perceptions of me were accurate	R	3.80	0.96	4	3.91	1.48	1–5	−0.76	0.31	0.04
24	It was difficult for me to express what I truly felt about my therapist ^R^	G	3.66	1.21	4	3.78	1.48	1–5	−0.65	−0.61	0.05

*Note.* Dim =  dimension; G =  Genuineness; MAD =  median absolute deviation; R =  Realism; SD =  standard deviation; SE =  standard error; Trimmed =  an average (mean) calculated after “trimming” a specified percentage of the smallest and largest data points*.* Items 3, 6, 8, 12, 14, 21, 22, and 24 were reversed before analysis.

^a^Statistically significant difference was observed in scores for in-person *vs.* remote settings (*t*-value =  −2.76, *p* <  0.01).

^R^=  reversed items.

### Preliminary analysis

Preliminary analyzes confirmed the suitability of the data for factor analysis, evidenced by a Kaiser-Meyer-Olkin measure of 0.977 and a significant Bartlett test of sphericity of χ²_[276]_ =  13392, *p* <  0.001.

### 24-item real relationship inventory—client form

#### Comparison of factor models.

A CFA was conducted to test the two-factor model using the item assignments hypothesized by the authors of the scale. The fit indices did not meet the commonly accepted thresholds for a good fit: χ^2^_[251]_ =  1772.733, *p* <  0.001, CFI =  0.886, TLI =  0.874, RMSEA =  0.093 [0.089–0.097], SRMR =  0.050. Both the Genuineness and the Realism factors showed statistically significant and relatively high factor loadings, indicating a moderate to strong association between the latent factors and their respective items. The covariation between these factors was estimated at.991, suggesting a very high degree of overlap between them. Additionally, Spearman’s rank-order correlation using the observed scores was 0.912.

Based on the results of the two-factor model, a one-factor model was tested. It exhibited a poor fit to the data: χ^2^_[252]_ =  1779.427, *p* <  0.001, CFI =  0.885, TLI =  0.874, RMSEA =  0.093 [0.089–0.097], SRMR =  0.050.

Two two-factor ESEM were fitted, including Geomin and targeted rotation. Both models showed similar fit indices (target rotation: χ^2^_[231]_ =  647.322, *p* <  0.001, CFI =  0.998, TLI =  0.998, RMSEA =  0.051 [0.046–0.055], SRMR =  0.034; and Geomin rotation: χ^2^_[231]_ =  642.032, *p* <  0.001, CFI =  0.998, TLI =  0.998, RMSEA =  0.050 [0.046–0.055], SRMR =  0.034).

In both ESEM models, all items showed significant loadings on the first latent factor (Genuineness), with estimates in the latest model ranging from 0.608 (item 14) to 0.886 (item 15), all with *p* <  0.000. This consistency underscores the robust relationship between these items and the Genuineness factor. A key difference emerged in the loadings on the second latent factor (Realism). While most items displayed significant loadings in both models, the magnitude and significance of these loadings varied. In the model based on the Geomin rotation, items like 16 (Estimate =  0.008, *p* =  0.735) and 23 (Estimate =  0.032, *p* =  0.201) maintained their non-significant loading on the Realism factor, akin to the previous model. However, item 12 showed a small yet non-significant loading (Estimate =  0.040, *p* =  0.198), deviating from its earlier insignificance. Another notable aspect was the covariance between the two latent factors. The covariance was slightly higher in the based on the Geomin rotation (0.119, *p* =  0.001) compared to the one based on the target rotation (0.109, *p* = .002), suggesting a marginally stronger relationship between the factors under the first rotation method. These large cross-loadings indicate the existence of a global factor or bifactor structure [39].

Lastly, a bifactor model with a general factor of the real relationship and two specific factors of Genuineness and Realism was tested using both CFA and ESEM approaches. The CFA model demonstrated satisfactory indices of fit: χ^2^_[228]_ =  1225.965, *p* <  0.001, CFI =  0.925, TLI =  0.909, RMSEA =  0.079 [0.075–0.083], SRMR =  0.043. The ESEM bifactor model showed good indices of fit: χ^2^_[210]_ =  482.464, *p* <  0.001, CFI =  0.999, TLI =  0.998, RMSEA =  0.043 [0.038–0.048], SRMR =  0.020.

The ancillary indices further supported the bifactor structure, with the general factor demonstrating Omega Hierarchical =  0.963 and a high explained common variance =  0.917, indicating that the general factor accounts for the bulk of the common variance, thereby supporting the bifactor model’s appropriateness.

### Measurement invariance testing between in-person and remote session formats

#### Multi-group confirmatory factor analysis.

In assessing the 24-item RRI-C scale, we began by examining configural invariance across in-person (*n* =  272) and remote session (*n* =  428) groups. The analysis of configural invariance revealed a strong fit, indicated by a CFI of 0.995, TLI of 0.994, RMSEA of 0.085, and SRMR of 0.061. This suggests a consistent factor structure across both groups, confirming that the pattern of how items load onto factors is uniform. Transitioning to metric invariance, a notable shift was observed. The model fit experienced a slight decrease, with ΔCFI at -0.024, ΔTLI at -0.025, and ΔRMSEA at -0.006, while ΔSRMR remained unchanged at 0.061. This shift hints at potential discrepancies in how items are weighted across groups, suggesting a need for further scrutiny. Further analysis was conducted on scalar invariance, which examines the equivalence of item intercepts across groups. Here, we observed additional reductions in model fit: ΔCFI decreased by 0.013, ΔTLI by 0.008, and RMSEA increased from 0.079 to 0.090, while SRMR stayed at 0.057. This change indicates potential variances in how items are interpreted across the in-person and remote groups. Finally, chi-square difference tests comparing the free model with both the weak and strong models provided significant insights. The weak vs. free model comparison was significant (*p* =  0.0021), favoring the free model, while the strong vs. free model comparison did not show a significant difference (*p* =  0.27). In conclusion, while our analysis confirms configural invariance, it raises questions about metric and scalar invariance. These findings suggest that the measurement and operationalization of constructs may differ between the two groups, warranting a closer examination.

#### Incorporating MIMIC model analysis.

To further investigate measurement invariance and account for demographic factors, a MIMIC model was utilized to test for invariance adjusted for age and gender (two categories: woman and man) between assessment types. The robust χ^2^ difference test using the Satorra-Bentler correction indicated that the addition of age and gender as covariates did not significantly alter the model fit, with a χ^2^ difference of 92, Δ*df* =  92, and *p* =  0.60. This non-significant result suggests that age and gender do not significantly impact the measurement structure across in-person and remote sessions, reinforcing the notion of invariance across these demographic variables.

#### Effect sizes of item bias .

The effect sizes of item bias, indicated by *d*_MACS_, were calculated to identify items contributing significantly to mismatches in factor models across different session formats and to estimate the magnitude of these mismatches. Items 3 and 4, with *d*_MACS_ values of 0.173 and 0.155 respectively, were identified as the most problematic, exerting the greatest impact on both metric and scalar variance. Similarly, items 7, 8, 9, 17, and 18 also displayed relatively high *d*_MACS_ values (0.128, 0.124, 0.124, 0.121, and 0.136, respectively), suggesting a pronounced disparity in their factor loading between the groups. The remaining items demonstrated minimal differences.

#### Multi-group alignment analysis.

Since we failed to establish metric and scalar invariance of the *24-item RRI-C* using multigroup CFA, we employed the multigroup alignment approach to compare the latent factor means. The *R*² values for loadings and intercepts were 0.998 and 1, respectively. This signifies a near-perfect replication of factor loadings and intercepts across groups, indicating that both the strength and the baseline of the measured constructs were consistent. In the alignment of lambda parameters (factor loadings), we found complete invariance across all items. With a parameter tolerance value set at 0.4, there was 0% non-invariance in item parameters. This indicates that all item factor loadings remained invariant, maintaining their consistency across different groups. Similarly, in the alignment of item intercepts, the analysis upheld invariance with a parameter tolerance value of 0.2. Again, across all items, there was 0% non-invariance in item parameters. This suggests that the intercepts of the items were as invariant as the loadings, with no significant deviation observed between groups. Joint item parameters, both lambda and nu, were consistent across groups. This lack of differential item functioning effects further reinforces the stability of our model. In summary, these results indicate a robust invariance in the 24-item RRI model, with no significant deviation in either item factor loadings or intercepts across groups. The high *R*² values and the absence of non-invariance in both loadings and intercepts suggest that any non-invariance in the model is negligible.

#### Item response theory.

Table 3 shows the values of the discrimination (*a*) and difficulty (*β*) parameters for the RRI-C total scale, respectively. S2 Fig displays item option characteristic curves and reliability. S1 Material provides a brief narrative description of the IRT findings.

**Table 3:** Item option characteristics for the RRI-C scales based on IRT models.

		24-item RRI-C					12-item RRI-C				
	Items	*α*	β1	β2	β3	β4	*α*	β1	β2	β3	β4
1	I was able to be myself with my therapist	2.37	−1.95	−1.51	−1.03	0					
2	My therapist and I had a realistic perception of our relationship	2.52	−2.03	−1.54	−0.9	0.25					
3	I was holding back significant parts of myself ^R^	1.9	−1.95	−1.04	−0.45	0.64					
4	I appreciated being able to express my feelings in therapy	3.11	−2.01	−1.56	−1.08	−0.07					
5	My therapist liked the real me	3.21	−2.19	−1.63	−0.47	0.57	3.26	−2.14	−1.61	−0.48	0.57
6	It was difficult to accept who my therapist really is ^R^	2.32	−2.22	−1.54	−0.96	0.11					
7	I was open and honest with my therapist	2.77	−2.09	−1.47	−0.98	0.23	2.22	−2.22	−1.58	−1.06	0.25
8	My therapist’s perceptions of me seem colored by his or her own issues ^R^	1.78	−2.35	−1.37	−0.89	0.08					
9	The relationship between my therapist and me was strengthened by our understanding of one another	2.48	−2.27	−1.55	−0.5	0.73					
10	My therapist seemed genuinely connected to me	3.11	−2.14	−1.29	−0.62	0.53	3.58	−2.04	−1.25	−0.61	0.51
11	I was able to communicate my moment-to-moment inner experience to my therapist	2.34	−2.47	−1.28	−0.58	0.68					
12	My therapist was holding back his/her genuine self ^R^	1.66	−2.93	−1.63	−0.66	0.71	1.72	−2.86	−1.6	−0.66	0.7
13	I appreciated my therapist’s limitations and strengths	2.49	−2.65	−1.71	−0.65	0.69	2.52	−2.6	−1.69	−0.66	0.69
14	We do not really know each other realistically ^R^	1.56	−2.03	−0.75	0.06	1.52	1.75	−1.91	−0.71	0.05	1.43
15	My therapist and I were able to be authentic in our relationship	3.94	−2.16	−1.34	−0.63	0.58	3.97	−2.1	−1.34	−0.65	0.57
16	I was able to see myself realistically in therapy	2.8	−2.49	−1.4	−0.72	0.61					
17	My therapist and I had an honest relationship	4.53	−2.07	−1.32	−0.81	0.32					
18	I was able to separate out my realistic perceptions of my therapist from my unrealistic perceptions	1.79	−3.01	−1.83	−0.73	0.83					
19	My therapist and I expressed a deep and genuine caring for one another	1.95	−2.24	−1.15	−0.03	1.19	2.25	−2.1	−1.09	−0.03	1.12
20	I had a realistic understanding of my therapist as a person	1.92	−2.43	−1.47	−0.65	1.06	2.05	−2.34	−1.43	−0.64	1.03
21	My therapist did not see me as I really am ^R^	3.24	−1.87	−1.19	−0.66	0.48	3.05	−1.88	−1.21	−0.68	0.48
22	I felt there was a significant holding back in our relationship ^R^	3.11	−1.96	−1.09	−0.54	0.44	3.03	−1.96	−1.1	−0.55	0.44
23	My therapist’s perceptions of me were accurate	2.89	−2.53	−1.47	−0.56	0.83	2.88	−2.48	−1.46	−0.58	0.83
24	It was difficult for me to express what I truly felt about my therapist ^R^	2.15	−1.98	−1.05	−0.46	0.66					

*Note.* ^*R*^ =  reversed items. Items 3, 6, 8, 12, 14, 21, 22, and 24 were reversed before analysis.

Overall, the range of discrimination parameters across the items of the scale suggests its general effectiveness in differentiating individuals with varying levels of the underlying trait, which contributes to the discriminative validity of the scale. Furthermore, the significant jumps in trait levels for some items, as seen in the *β*-parameters of items like Item 19 and Item 14, could indicate potential issues with the response options or their interpretation by respondents. These aspects can lead to inaccuracies or misinterpretations in scoring and interpreting these items, which requires careful consideration in the application of the scale.

#### Internal consistency and reliability.

The reliability analysis of the 24-item RRI-C full scale revealed good internal consistency: Cronbach’s alpha =  0.97, McDonald’s omega total =  0.97, and average item correlations =  0.55. Additionally, the total scale demonstrated IRT marginal reliability greater than.80 within the range of theta values between -3.7 and + 2. Table 4 presents the reliability estimates.

**Table 4:** Descriptive statistics, internal consistency reliability, precision, and inter-scale correlations.

Descriptive statistics	24-item	12-item
Potential Range	24 to 120	12 to 60
Observed Range	25 to 120	12 to 60
Mean, *SD*	92.5 (19.50)	45.3 (9.90)
Skew	−1.01	−0.81
Kurtosis	0.82	0.44
Standard Error of Measurement (SE_m_)	3.38	2.42
Standard Error of Difference (SE_d_)	4.78	3.43
Internal consistency reliability		
Average inter-item *r*	0.55	0.57
Cronbach’s α	0.97	0.94
McDonald’s ω total	0.97	0.95
Clinical change benchmarks		
90% Critical Change	5.56	3.99
95% Critical Change	6.62	4.75
Minimal Important Difference (MID)	9.75	4.95
Minimum Change for a Reliable Change	9.36	6.72

Note. MID was operationally defined as *d* =  0.5.

The Genuineness and Realism subscales showed Cronbach’s alpha values of 0.94 and 0.93, McDonald’s omega total values of 0.94 and 0.93, and average item correlations of 0.56 and 0.53, respectively.

#### Scale and subscale correlations.

The 24-item RRI-C demonstrated very strong positive correlations with the Genuineness (*r* =  0.98, *p* <  0.001) and Realism (*r* =  0.98, *p* <  0.001) subscales. The Genuineness and Realism subscales were strongly correlated (*r* =  0.93, *p* <  0.001).

#### Associations between sociodemographic, clinical, and treatment variables and scale scores.

Table 5 provides detailed correlation coefficients for the RRI total scale. The average absolute correlations between the demographic, clinical, and treatment subsets of variables were very weak.

**Table 5:** Criterion validity correlations with patient demographics, diagnoses, and objective therapy characteristics.

Criterion Variable	24-item	12-item	Cohen’s *q*
Age	.05	.06	.00
Biological sex	−.05	−.04	.00
Gender	−.04	−.02	−.01
Education	.07	.08	.00
Ethnicity	−.06	−.06	.00
*Average absolute correlation across subset*	.05	.05	.00
Any psychiatric disorder	.05	.05	.00
Any anxiety disorder	−.00	.00	.00
Any (unipolar) depressive disorder	.06	.06	.00
Any trauma- and stressor-related disorders	.04	.06	−.01
Any neurodevelopmental disorder	.02	.04	−.01
Any bipolar or related disorder	.00	.00	.00
Any eating disorder	−.05	−.04	.00
Any disruptive behavior and dissocial disorder	−.03	−.03	.00
Schizophrenia or any other psychotic disorders	−.11*	−.11*	.00
Any cluster A personality disorder	.03	.02	.00
Any cluster B personality disorder	−.00	−.00	.00
Any cluster C personality disorder	.06	.06	.00
*Average absolute correlation across subset* ^c^	.04	.04	.00
Therapy length (months, ordinal; see prior table)	.15***	.17***	−.01
Session frequency (ordinal, see prior table)	.13**	.14***	.00
Session attendance	.03	.03	.00
Therapy location	−.04	−.05	.00
Therapist’s sex	−.03	−.00	−.01
*Average absolute correlation across subset*	.08	.08	.00

*Note.* Coefficients are point-biserial correlations for dichotomized variables, point-biserial correlations for dummy-coded categorical variables, Spearman correlations for ordinal variables, and Spearman correlations for continuous variables.

Cohen’s *q* compares the scores of the 24-item and 12-item versions of the RRI-C.

^c^“Any psychiatric disorder” excluded from the matrix.

*p < .05, **p < .01, ***p < .001.

#### Evaluation of criterion validity.

Table 6 displays all the calculated correlation coefficients. The full form of the RRI-C demonstrated minimal associations with all measures related to the personality traits of the patients (BFI-2-XS and LPFS-BF 2.0) and their current mental health status (PHQ-9, GAD-7, I-PANAS-SF and three single item scales). On the other hand, it exhibited moderate associations with other measures assessing elements of the therapeutic relationship (WAI-SR, SPARQ, and PSQ items reflecting the degree to which the issue encountered in the session was dealt with and resolved within the same session). Additionally, it showed a moderate correlation with a measure of session outcome (SES). The pattern of findings suggests responses were more associated with session features than personality or dispositional response sets.

**Table 6:** Criterion validity correlations between the 24-item and 12-item RRI-C and validated scales.

Criterion Variable	*Mean (SD)*	24-item	12-item	Cohen’s *q*
Trait measures				
BFI-2-XS agreeableness	11.40 (2.39)	.19***	.20***	.00
BFI-2-XS conscientiousness	9.60 (3.01)	.07	.07	.00
BFI-2-XS extraversion	8.21 (3.02)	.14***	.14***	.00
BFI-2-XS negative emotionality	1.90 (2.83)	−.06	−.07	.00
BFI-2-XS open-mindedness	11.40 (2.46)	.16***	.13***	.01
LPFS-BF 2.0 total score	27.90 (7.32)	−.24***	−.22***	−.01
LPFS-BF 2.0 self-functioning	15.20 (4.35)	−.21***	−.20***	.00
LPFS-BF 2.0 interpersonal functioning	12.70 (3.95)	−.21***	−.19***	−.01
*Average absolute correlation across subset* ^a^		.15	.14	.00
State measures				
GAD-7	1.00 (6.10)	−.13***	−.11***	−.01
I-PANAS-SF negative affect	11.60 (4.02)	−.19***	−.16***	−.01
I-PANAS-SF positive affect	14.60 (4.14)	.22***	.21***	.00
PHQ-9	9.56 (6.24)	−.18***	.16***	−.15
SI - Psychosocial functioning	1.81 (1.07)	−.10^*^	−.09^*^	.00
SI - Quality of life	1.79 (.85)	−.17***	−.16***	.00
SI - Symptom severity	2.33 (.92)	−.07	−.06	.00
*Average absolute correlation across subset*		.15	.14	.00
Therapeutic relationship measures				
PSQ (yes) *% (F)*	16% (112)	−.27***	−.28***	.00
SPARQ Positive Affect	12.26 (3.30)	.67***	.67***	.00
SPARQ Negative Affect	3.18 (3.13)	−.51***	−.47***	−.02
RISQ	.15 (.55)	−.33***	−.32***	.00
WAI-SR total score	4.50 (13.10)	.68***	.67***	.01
WAI-SR goal	13.60 (4.93)	.61***	.60***	.01
WAI-SR task	12.50 (4.80)	.60***	.57***	.02
WAI-SR bond	14.50 (4.61)	.66***	.67***	−.01
*Average absolute correlation across subset* ^b^		.51	.50	.01
Session outcome measure				
SES	4.06 (.84)	.62***	.58***	.03

*Note.* BFI-2-XS =  Big Five Inventory–2-Extra-Short form; GAD-7 =  Generalized Anxiety Disorder-7; G =  Genuineness subscale; I-PANAS-SF =  International Positive and Negative Affect Schedule - Short Form; LPFS =  Level of Personality Functioning Scale; PHQ-9 =  Patient Health Questionnaire-9; PSQ =  Post-Session Questionnaire; R =  Realism subscale; RISQ =  Rift In-Session Questionnaire; SES =  Session Evaluation Scale; SI =  Single-item global measures of symptom severity, psychosocial functioning, and quality of life; SPARQ =  in-Session Patient Affective Reactions Questionnaire; WAI-SR =  Working Alliance Inventory – Short Revised.

Coefficients are Spearman correlations.

Cohen’s *q* compares the scores of the 24-item and 12-item versions of the RRI-C.

^a^LPFS-BF 2.0 total score excluded from the matrix.

^b^PSQ (yes) and WAI-SR subscores excluded from the matrix.

*p < .05, **p < .01, ***p < .001.

### 12-item real relationship inventory—client form

#### Comparison of factor models.

The two-factor CFA results showed the following indices of fit: a significant chi-squared value: χ^2^_[53]_ =  433.925, *p* <  0.001, CFI =  0.935, TLI =  0.919, RMSEA =  0.101 [0.093–0.110], SRMR =  0.042.

A one-factor model was also tested and exhibited a poor fit to the data: χ^2^_[54]_ =  437.739, *p* <  0.001, CFI =  0.934, TLI =  0.920, RMSEA =  0.101 [0.092–0.110], SRMR =  0.042.

Two two-factor ESEM were fitted, including Geomin and targeted rotation. Both models showed similar fit indices (target rotation χ^2^_[45]_ =  111.916, *p* <  0.001, CFI =  0.999, TLI =  0.998, RMSEA =  0.046 [0.035–0.057], SRMR =  0.028; and Geomin rotation: χ^2^_[45]_ =  110.014, *p* <  0.001, CFI =  0.999, TLI =  0.999, RMSEA =  0.045 [0.035–0.056], SRMR =  0.028.

In both ESEM models, all items showed significant loadings on the first latent factor (Genuineness), with estimates in the Geomin rotation model ranging from 0.664 (item 14) to 0.891 (item 21), and in the Targeted rotation model ranging from 0.669 (item 14) to 0.886 (item 21), all with *p* <  0.000. This consistency underscores the robust relationship between these items and the Genuineness factor. A key difference emerged in the loadings on the second latent factor (Realism). While most items displayed significant loadings in both models, the magnitude and significance of these loadings varied. In the Geomin rotation model, item 20 showed a significant loading (Estimate =  0.209, *p* <  0.000), whereas in the Targeted rotation model, its loading was slightly lower yet still significant (Estimate =  0.204, *p* <  0.000). In contrast, item 12 showed a non-significant loading in both the Geomin (Estimate =  -0.018, *p* =  0.575) and Targeted (Estimate =  -0.023, *p* =  0.484) rotation models. Another notable aspect was the covariance between the two latent factors. The covariance was slightly higher in the Geomin rotation model (0.131, *p* <  0.000) compared to the Targeted rotation model (0.116, *p* =  0.001), suggesting a marginally stronger relationship between the factors under the Geomin rotation method.

Lastly, a bifactor model with a general factor of the real relationship and two specific factors of genuineness and realism was fitted. The CFA model showed acceptable indices of fit: χ²_[42]_ =  286.231, *p* <  0.001; CFI =  0.958; TLI =  0.934; RMSEA =  0.091 (90% CI [0.081, 0.101]); and SRMR =  0.035. The ESEM model showed excellent the following indices of fit: χ^2^_[45]_ =  111.916, *p* <  0.001, CFI =  0.999, TLI =  0.998, RMSEA =  0.046 [0.035–0.057], SRMR =  0.028.

The ancillary indices further supported the bifactor structure, with the general factor demonstrating Omega Hierarchical =  0.940 and a high explained common variance =  0.923.

### Measurement invariance testing across in-person and remote session formats

#### Multi-group confirmatory factor analysis.

The analysis of configural invariance of the 12-item RRI-C showed a robust fit, indicated by a CFI of 0.997, TLI of 0.996, RMSEA of 0.073, and SRMR of 0.046 in the free model. This suggests a consistent factor structure across the two groups, confirming uniformity in how items load onto factors. Transitioning to metric invariance, a notable shift was observed. The fit of the model experienced a slight decrease, with a CFI of 0.996, TLI of 0.996, RMSEA of 0.080, and SRMR of 0.051 in the weak invariance model. The changes in CFI and TLI were -0.001 and 0.000, respectively, ΔRMSEA was + 0.007, and ΔSRMR was + 0.005, indicating potential discrepancies in how items are weighted across groups. Further analysis on scalar invariance revealed additional reductions in model fit: ΔCFI decreased by -0.001, ΔTLI remained unchanged, and RMSEA increased from 0.080 to 0.089, while SRMR decreased slightly to 0.047 in the strong invariance model. These changes suggest potential variances in item interpretation across groups. Finally, chi-square difference tests comparing the free model with both the weak and strong models provided significant insights. The weak vs. free model comparison showed a significant difference with a chi-square difference of 32.3 (*p* <  0.001), favoring the free model. However, the strong *vs.* free model comparison did not show a significant difference with a chi-square difference of 55.4 (*p* =  0.12), indicating that the factor structure and item loading pattern are the same, but the item means (and thus the scale scores) may be different.

#### Incorporating MIMIC model analysis.

The MIMIC model was used to test for invariance adjusted for age and gender across assessment types. The robust χ² difference test indicated that adding age and gender as covariates did not significantly alter the model fit: χ² difference =  34.1, Δ*df* =  44, *p* =  0.86.

#### Effect sizes of item bias.

The effect sizes of item bias were calculated. Item 14 exhibited the highest disparity with a *d*_MACS_ value of 0.1549, indicating a substantial impact on metric and scalar variance. Item 7 followed with a *d*_MACS_ of 0.1281, suggesting notable variance in factor loading. Item 15 (*d*_MACS_ =  0.0929) and item 23 (*d*_MACS_ =  0.0615) showed moderate disparities. Items 12 and 13 both recorded *d*_MACS_ values of 0.0515, and item 21 had 0.0418, indicating some differences in factor loading between groups. Items 5, 10, 19, 20, and 22 demonstrated lower *d*_MACS_ values (0.0677, 0.0149, 0.0186, 0.0221, and 0.0141, respectively), reflecting minimal factor loading differences across the groups.

#### Multi-group alignment analysis.

In the 12-item RRI-C multigroup alignment, loadings and intercepts showed high consistency with *R*² values of 0.998 and 1 respectively, indicating uniformity in both factor strength and baseline between groups. Complete invariance was observed in lambda parameters (factor loadings) with a parameter tolerance of 0.4, leading to 0% non-invariance in item parameters. This result was mirrored in the nu parameters (item intercepts) with a tolerance value of 0.2, also showing 0% non-invariance. Items such as rri5 (0.807, 3.84), rri7 (0.761, 4.06), and rri10 (0.880, 3.86) maintained consistent loadings and intercepts between groups. The absence of differential item functioning effects (0 for all items in both groups) further validates the stability of the model. The findings confirm robust invariance in the RRI model, with minimal deviations between different session formats.

#### Item response theory.

Table 3 shows the values of the discrimination (*a*) and difficulty (*β*) parameters for the 12-item RRI-C, respectively. S3 Fig displays item option characteristic curves and reliability. S1 Material provides a brief narrative description of the IRT findings.

The 12-item RRI-C also demonstrated substantial variability in both discrimination power and difficulty parameters. The range in difficulty parameters across items suggested that the scale can assess a broad spectrum of the trait. However, the differences in the discrimination parameters between the items indicated that while some items were highly effective in distinguishing between trait levels, others might be less effective, potentially impacting the overall discriminative power of the scale. Overall, the IRT results highlight the nuances in item functioning within the scale, underscoring the importance of considering both discrimination and difficulty parameters in evaluating and interpreting the scale’s effectiveness.

#### Reliability and item analysis.

Projected and observed internal consistency were both Cronbach’s alpha =  0.94. McDonald’s omega total was 0.95, while average inter-item was *r* =  0.57. Additionally, the total scale demonstrated IRT marginal reliability greater than 0.80 within the range of theta values between -3.3 and + 1.8. Table 4 presents the reliability estimates.

The Genuineness and Realism subscales showed Cronbach’s alpha values of 0.89 and 0.88, McDonald’s omega total values of 0.99 and 0.89, and average item correlations of 0.57 and 0.55, respectively.

#### Retained content coverage, agreement, and bias.

Projected correlation between the 12-item and 24-item versions of the RRI-C was *r* =  0.91. The observed correlation was *r* =  0.98 (*p* <  0.001). The 12-item RRI-C showed robust correlations with its subscales (*r* =  0.98, *p* <  0.001, in both cases). These short subscales were also very strongly correlated (*r* =  0.91, *p* <  0.001). This is consistent with the strong general factor in the bifactor model. S1 Table presents correlation coefficients for the 24-item and 12-item RRI-C scales and subscales, along with Steiger’s *z*-test results.

Cohen’s *q* analyzes were also performed to test the difference in criterion correlations observed with the long vs. short forms with all demographics, diagnoses, and objective therapy characteristics (see Table 5), as well as with personality traits, current mental health status, therapeutic relationship and session outcome variables (see Table 6). The range of Cohen’s *q* values obtained was between -0.09 and 0.05, indicating a minimal effect size in all investigated categories. Importantly, none of these findings reached statistical significance.

Bland-Altman plots and regressions were used to investigate the agreement between the abbreviated and comprehensive versions of the scales (see S4 Fig). These versions were adjusted to have identical scaling, allowing for direct comparison (potential scores of 24-120 for the comprehensive version and 12-60 for the subscales scores). Upon comparison, the total score reflected an average discrepancy of 1.85 points higher for the comprehensive version. This variation is statistically insignificant (*p* =  0.12) with a slope of -0.013. The discrepancy was nearly zero within the score range where most of the participants scored less. These results support the conclusion that there is strong agreement and negligible bias between the abbreviated and the comprehensive version.

#### Score precision.

Table 4 provides the measurements of standard error of measurement (SE_m_) and difference (SE_d_), which indicate the impact of measurement error on score deviation for both the 24-item and 12-item versions of the RRI-C. It also presents critical change values for 90% and 95% confidence levels, indicating thresholds for significant changes beyond measurement error, the minimally important difference (MID), representing the smallest score change considered beneficial, and the minimum change for a reliable change benchmark threshold [64] to identify statistically reliable changes.

## Discussion

The purpose of this article was to assess the psychometric properties of the 24- and 12-item versions of the RRI-C. Our results align with a bifactor structure with two dimensions as hypothesized by the RRI-C developers for both the set of complete items and the version of 12 items based on the results of the validation study [17,18].

### Factor structure

The CFAs and the ESEMs conducted on the RRI-C in both its 24-item and 12-item forms provided valuable insights into their factor structures. The results of these analyzes revealed a robust psychometric property with the 24- and 12-item versions of the RRI-C exceeding widely accepted thresholds for a good fit in structural models [65].

For the 24-item RRI-C, both the Geomin and targeted rotation ESEM models exhibited strong fit indices. In both models, all items demonstrated significant loadings on the “Genuineness” factor, reinforcing the strong relationship between these items and the Genuineness factor. Interestingly, the covariance between the Genuineness and Realism factors was slightly higher in the Geomin rotation model, suggesting a stronger relationship under this rotation method. The bifactor ESEM model further underscored this robustness, showing excellent fit indices.

The 12-item RRI-C also displayed promising results. Both the Geomin and targeted rotation ESEM models demonstrated similar fit indices. All items had significant loadings on the Genuineness factor in both models, indicating a consistent and strong relationship. The covariance between Genuineness and Realism was slightly higher in the Geomin rotation model. The bifactor model for the 12-item version also demonstrated good fit indices, echoing the patterns observed in the 24-item version.

The strong performance of the bifactor models in both versions aligns with the possibility of a global construct, as suggested by the developers of the scale [17]. These results indicate that both the 24- and 12-item versions of the RRI-C possess sound factor structures, with the bifactor models particularly demonstrating a robust fit. Notably, a shortened 8-item version of the RRI-C [66] has recently been developed using best practices for creating abbreviated versions of psychological assessments (including exploratory and confirmatory factor analyses on independent samples, as well as item response theory) and has demonstrated excellent psychometric properties and supports the bifactor model of the real relationship.

### Measurement Invariance between in-person and remote session formats

The MGCFA for the 24-item RRI-C indicated strong configural invariance across in-person and remote session groups. This consistency in factor structure between groups was a positive indicator. However, challenges emerged in metric and scalar invariance, with slight decreases in model fit. These shifts suggest potential discrepancies in item weighting and interpretation across groups. The scalar invariance analysis further reinforced these potential variances, as indicated by additional reductions in model fit. Despite these issues, the alignment analysis revealed a high degree of invariance in factor loadings and intercepts and complete invariance across all items. This suggests that the overall structure of the model remains robust across different session formats. Nonetheless, items like 3 (“I was holding back significant parts of myself”) and 4 (“I appreciated being able to express my feelings in therapy”), with higher *d*_MACS_ values, might contribute to mismatches in factor models, necessitating further scrutiny.

For the 12-item version, the MGCFA also showed robust configural invariance with strong fit indices in the free model. The transition to metric and scalar invariance indicated some potential discrepancies, although changes in fit were minor. The chi-square difference tests further suggested that the strong invariance model did not significantly differ from the free model. In line with the 24-item version, the alignment analysis for the 12-item RRI-C revealed high consistency in loadings and intercepts, with no non-invariance in item parameters. This indicates a stable model across session formats. Items like 14 (“We do not really know each other realistically”), showing higher *d*_MACS_ values, indicate notable variance in factor loading, warranting attention.

Both versions of the RRI-C demonstrate a high level of configural invariance, suggesting consistent factor structures across different session formats. While there are some challenges in metric and scalar invariance, particularly in the 24-item version, the overall structure and stability of the scale are affirmed by the alignment analysis. The differences observed in item functioning point to areas where the scale might benefit from refinement to enhance its applicability and interpretability across diverse settings. The findings from these analyses are crucial for informing future revisions and applications of the RRI-C, ensuring its effectiveness and reliability in different contexts.

### Item response analysis

The IRT analyses of the RRI-C provided insights into the discrimination and difficulty parameters of individual items in both the 24-item and 12-item versions, revealing the strengths and potential areas for improvement in the scale.

For the 24-item RRI-C, the discrimination parameters exhibited significant variability, reflecting the differing capacities of the items to differentiate respondents based on their trait levels. Notably, Item 17 (“My therapist and I had an honest relationship”) stood out with the highest discrimination parameter, demonstrating an exceptional ability to differentiate between levels of the trait. On the contrary, item 14 (“We do not really know each other realistically”) had the lowest discrimination parameter, indicating a lower, albeit still adequate, differentiation capacity. This variability in discrimination parameters underscores potential implications for the precision and effectiveness of subscales. In terms of difficulty parameters, there was a wide range across the scale. Disparities in item thresholds (see, for example, items 14 and item 19: “My therapist and I expressed a deep and genuine caring for one another”) could have implications for scoring and interpretation, potentially leading to inaccuracies or misinterpretations.

The 12-item RRI-C also demonstrated substantial variability in both discrimination power and difficulty parameters. The range in difficulty parameters across items suggested that the scale can assess a broad spectrum of the trait. However, the differences in the discrimination parameters between the items indicated that while some items were highly effective in distinguishing between trait levels, others might be less effective, potentially impacting the overall discriminative power of the scale.

Overall, the IRT results highlight the nuances in item functioning within both versions of the RRI-C. Furthermore, they suggested that the 24-item version, despite its broader range of discrimination and difficulty of items, might require refinement of certain items to enhance its precision. Meanwhile, the 12-item version, with its more effective measurement of differences in genuineness and realism traits, appears to offer a more concise yet robust alternative. However, careful consideration is still warranted for items with high difficulty parameters to ensure the effectiveness of the scale at different levels of the underlying traits.

### Reliability

Both versions of the RRI-C demonstrated excellent internal consistency. The Cronbach’s alpha and McDonald’s omega total values indicated strong reliability for the full and the short versions, speaking volumes to the homogeneity of the items in measuring the constructs of genuineness and realism.

### Content coverage, agreement, and bias

Strong evidence for comparable measurement capabilities was seen in the high correlation coefficients between the total scores of the RRI-C full length and shortened versions. Both versions of the RRI exhibited similar criterion correlations, displaying equivalent predictive capabilities for external variables. Cohen’s *q*, which reflects the difference between two correlation coefficients, underscored the comparability of the abbreviated version with the comprehensive one, and their equivalent performance in predicting relevant external measures. The differences were consistently in the range that would be considered small or clinically negligible [67].

The Bland-Altman analyzes revealed a high level of agreement between the scores derived from the full- and short versions. Minimal bias was observed, suggesting that the 12-item version does not systematically over- or under-estimate scores compared to the 24-item version.

Overall, the results suggest that the short version of the RRI-C can effectively replace the full version without a significant loss of precision or content coverage.

### Criterion validity

The criterion validity of both versions of the RRI was evidenced by minimal correlations with patient demographics, clinical and treatment variables, traits, and state measures, but substantial convergent correlations with measures that evaluated specific aspects of the therapeutic relationship and session outcomes. This pattern supports the ability of the scales to capture unique effects within therapy sessions, without significant influence of the general psychopathology of the patient or unrelated external factors. All versions moderately correlated with the working alliance, in line with theoretical expectations [5] and recent meta-analytic findings [8]. The RRI-C showed similar correlations with all three dimensions of the working alliance, somewhat inconsistent with the findings of the validation study [17] that found a stronger correlation with the bond dimension, but aligns with more recent research [68]. Furthermore, the short and full versions of RRI-C showed a moderate positive correlation with the SPARQ Positive Affect scale (assessing patient’s reaction to the therapist) and a moderate negative correlation with its Negative Affect Scale, which partially reflects the concept of negative transference. Weak negative correlations with alliance ruptures (SPQ) and therapeutic relationship rifts (RISQ) were observed.

### Limitations and future directions

Despite these promising results, our study is not without limitations. It is worth noting that, while our results indicate that the 12-item RRI can substitute the 24-item version without losing significant predictive accuracy, these findings may not extend to all demographic or clinical populations. Future research could focus on further refining the scales by reevaluating items with lower discrimination parameters or high difficulty parameters. The RRI-C-SF [66] is a promising first step in this direction. Additionally, investigating the high covariation between the Genuineness and Realism factors could provide further insight into the underlying constructs of these scales.

## Conclusions

In summary, our results support and bolster those of Kelley et al. (2010), indicating the potential existence of a bidimensional construct of the real relationship as measured by the RRI-C based on the strong correlation between the Genuineness and Realism scales. Findings also extend prior work by showing that the RRI-C versions have functionally identical reliability and factor structure when therapy is online *vs.* in-person. The short form also shows negligible bias in scores compared to the full length, further supporting its advantages in many applications.

## Supporting information

S1 Table Scales and subscales correlations and Steiger’s Z-test results. (DOCX)

S1 Fig Distribution of responses for each item across Likert scale levels: A histogram representation. (TIF)

S2 Fig Item option characteristic curves and reliability for the 24-item RRI-C total score. (TIF)

S3 Fig Item option characteristic curves and reliability for the 12-item RRI-C total score. (TIF)

S4 Fig Bland-Altman Plots comparing the accuracy of 24-item and 12-item versions of RRI-C form. (TIF)

S1 Material Narrative description of the IRT findings. (DOCX)
