# Supplementary material for: Evaluating the psychometric properties of the 24-item and 12-item real relationship inventory-client forms
Source: PLoS One. 2025 Mar 3;20(3):e0311411. doi: 10.1371/journal.pone.0311411 (PMC11875345; doi:10.1371/journal.pone.0311411)
Supplement: S1 Table — (DOCX) [file pone.0311411.s001.docx]

**S1 Table** Scales and subscales correlations and Steiger’s *z*-test results for dependent correlations

| Scale correlations ^a^ | 24-item | 24-item | 24-item | 12-item | 12-item | 12-item |
| --- | --- | --- | --- | --- | --- | --- |
|  | Total score | Genuineness | Realism | Total score | Genuineness | Realism |
| 24-item RRI total score | -- |  |  |  |  |  |
| 24-item RRI Genuineness score | .98 | -- |  |  |  |  |
| 24-item RRI Realism score | .98 | .93 | -- |  |  |  |
| 12-item RRI total score | .98 | .95 | .96 | -- |  |  |
| 12-item RRI Genuineness score | .96 | .96 | .92 | .98 | -- |  |
| 12-item RRI Realism score | .94 | .90 | .96 | .98 | .91 | -- |

Note. The correlations between the 24-item and 12-item versions of the RRI-C are displayed in the lower left section of the table.

All the *p*-values are < 0.001.

^a^ Steiger’s *z*-test comparing the 24-item to the 12-item versions of the RRI-C resulted in: *z* = 1.33, *p* = .182.
