# Supplementary figures and images for: Evaluating the psychometric properties of the 24-item and 12-item real relationship inventory-client forms
Source: PLoS One. 2025 Mar 3;20(3):e0311411. doi: 10.1371/journal.pone.0311411 (PMC11875345; doi:10.1371/journal.pone.0311411)

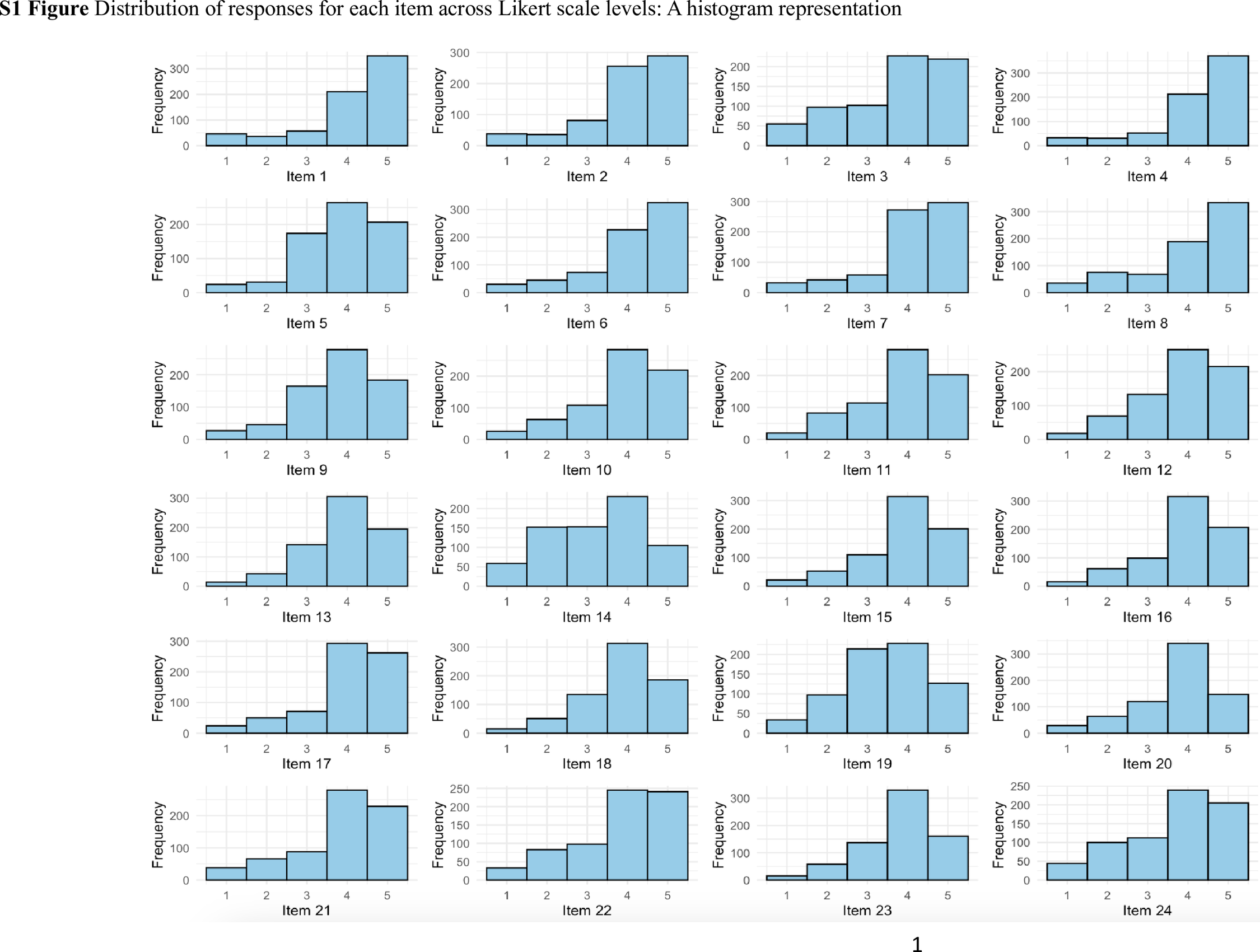

Supplement: S1 Fig — (TIF) [file pone.0311411.s002.tif]

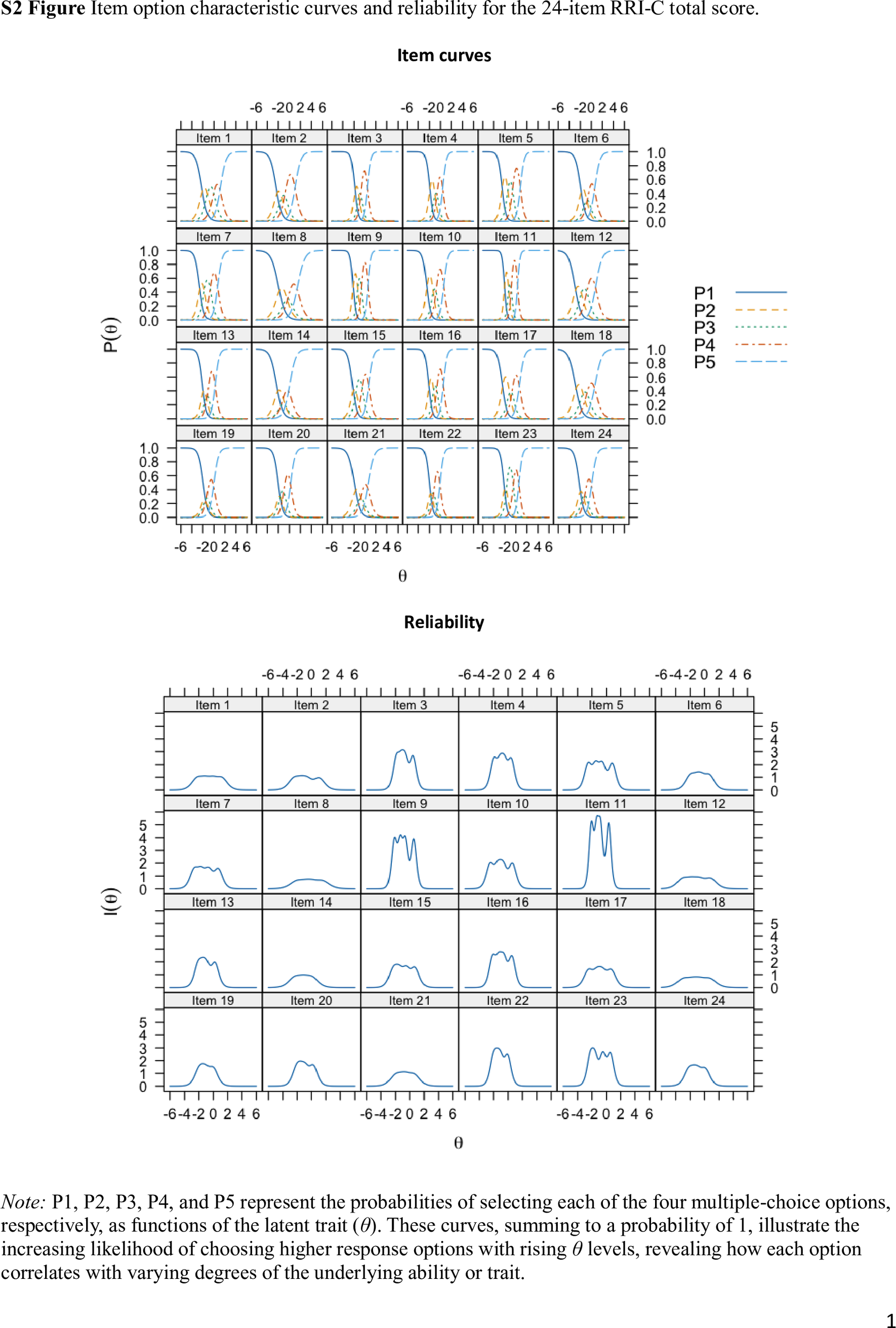

Supplement: S2 Fig — (TIF) [file pone.0311411.s003.tif]

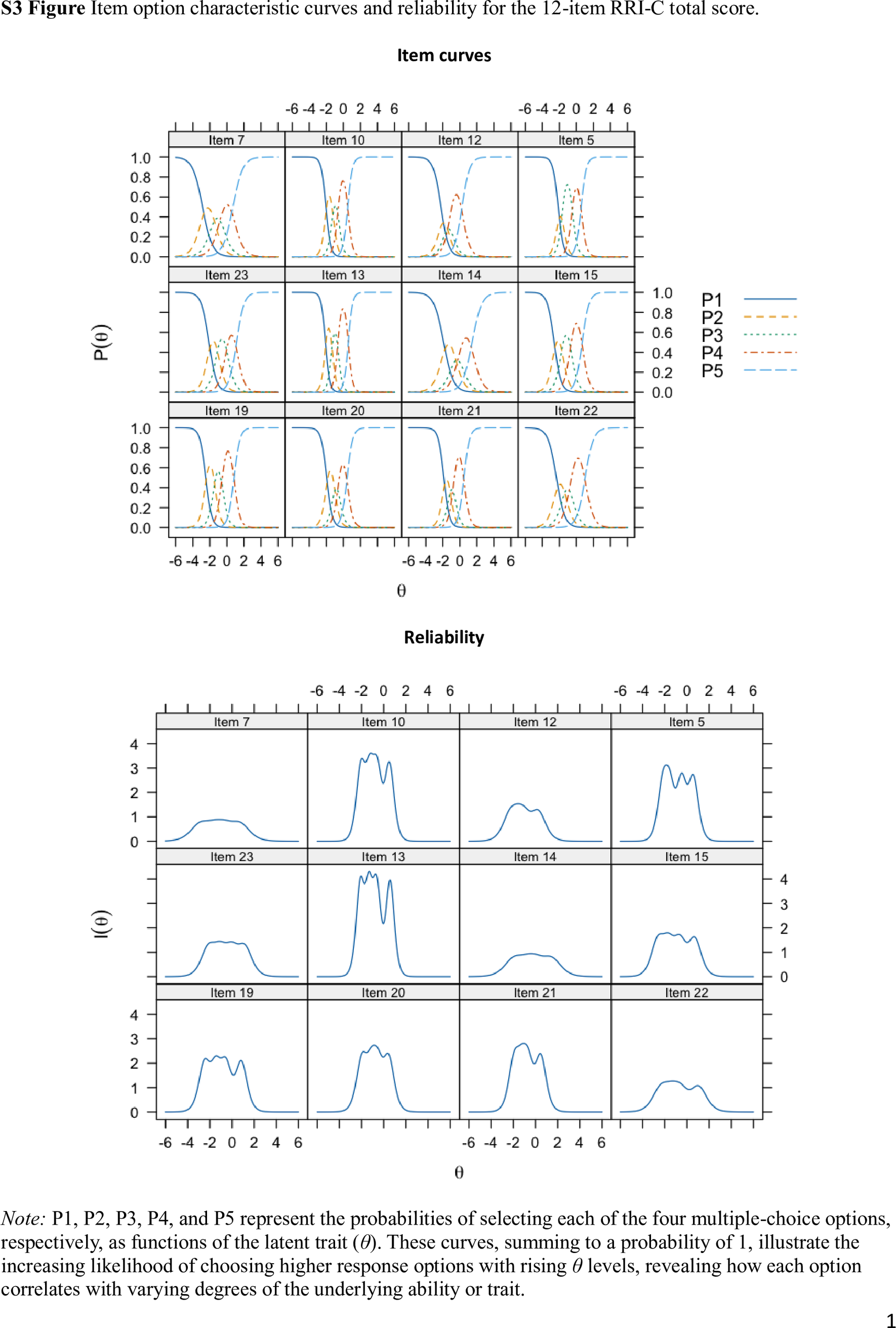

Supplement: S3 Fig — (TIF) [file pone.0311411.s004.tif]

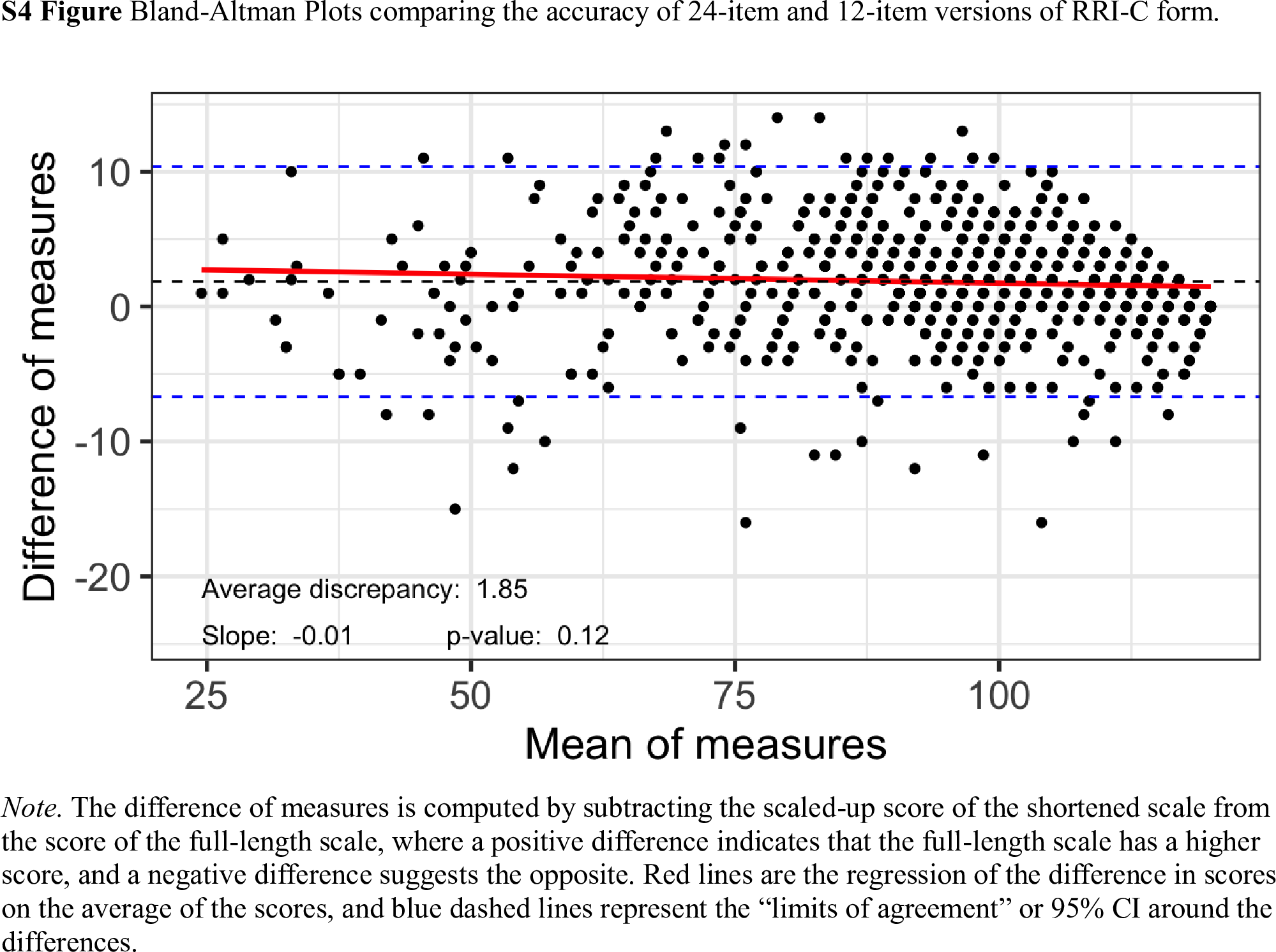

Supplement: S4 Fig — (TIF) [file pone.0311411.s005.tif]
