# Supplementary material for: Evaluating the psychometric properties of the 24-item and 12-item real relationship inventory-client forms
Source: PLoS One. 2025 Mar 3;20(3):e0311411. doi: 10.1371/journal.pone.0311411 (PMC11875345; doi:10.1371/journal.pone.0311411)
Supplement: S1 Material — (DOCX) [file pone.0311411.s006.docx]

**S5 Material.** Narrative description of the IRT findings.

**24-item RRI-C**

The discrimination parameters for the items varied significantly, reflecting their different capabilities in differentiating respondents based on their trait levels. For example, Item 17 showed the highest discrimination parameter at 4.53, indicating a very high capacity to differentiate between levels of the trait. On the contrary, item 14 had the lowest discrimination parameter of 1.56, suggesting a lower but still high (α = 1.35–1.69) differentiation capacity.

The item thresholds (βs), representing the difficulty parameters, also demonstrated a wide range across the scale. Notably, item 19 exhibited an unusual pattern in its β3 and β4 parameters (-0.03 and 1.19, respectively), indicating a considerable jump in trait level between these response categories. Such disparities could have implications for how scores are interpreted. Similarly, item 14 was notable for its substantial jump in trait level between the third and fourth response categories (0.06 and 1.52, respectively), which might reflect issues in how respondents perceive these response options. Overall, the range of discrimination parameters across the items of the scale.

**12-item RRI-C**

For the 12-item RRI-C, the discrimination parameters exhibited significant variability, reflecting the differing capacities of the items to differentiate respondents based on their trait levels. Notably, Item 17 (“My therapist and I had an honest relationship”) stood out with the highest discrimination parameter, demonstrating an exceptional ability to differentiate between levels of the trait. On the contrary, item 14 (“We do not really know each other realistically”) had the lowest discrimination parameter, indicating a lower, albeit still adequate, differentiation capacity. This variability in discrimination parameters underscores potential implications for the precision and effectiveness of subscales. In terms of difficulty parameters, there was a wide range across the scale. Disparities in item thresholds (see, for example, items 14 and item 19: “My therapist and I expressed a deep and genuine caring for one another”) could have implications for scoring and interpretation, potentially leading to inaccuracies or misinterpretations.
